# Spontaneous mutations in the single *TTN* gene represent high tumor mutation burden

**DOI:** 10.1038/s41525-019-0107-6

**Published:** 2020-01-14

**Authors:** Ji-Hye Oh, Se Jin Jang, Jihun Kim, Insuk Sohn, Ji-Young Lee, Eun Jeong Cho, Sung-Min Chun, Chang Ohk Sung

**Affiliations:** 1 Department of Medical Science, Asan Medical Institute of Convergence Science and Technology, University of Ulsan College of Medicine, Asan Medical Center, Seoul, Republic of Korea; 2 Center for Cancer Genome Discovery, Asan Institute for Life Science, University of Ulsan College of Medicine, Asan Medical Center, Seoul, Republic of Korea; 3 Department of Pathology, Asan Medical Center, University of Ulsan College of Medicine, Seoul, Republic of Korea; 4 Biostatistics and Clinical Epidemiology Center, Research Institute for Future Medicine, Samsung Medical Center, Seoul, Republic of Korea

**Keywords:** Cancer genomics, Molecular medicine

## Abstract

Tumor mutation burden (TMB) is an emerging biomarker, whose calculation requires targeted sequencing of many genes. We investigated if the measurement of mutation counts within a single gene is representative of TMB. Whole-exome sequencing (WES) data from the pan-cancer cohort (*n* = 10,224) of TCGA, and targeted sequencing (tNGS) and *TTN* gene sequencing from 24 colorectal cancer samples (AMC cohort) were analyzed. *TTN* was identified as the most frequently mutated gene within the pan-cancer cohort, and its mutation number best correlated with TMB assessed by WES (rho = 0.917, *p* < 2.2e-16). Colorectal cancer was one of good candidates for the application of this diagnostic model of TTN-TMB, and the correlation coefficients were 0.936 and 0.92 for TMB by WES and TMB by tNGS, respectively. Higher than expected *TTN* mutation frequencies observed in other FLAGS (FrequentLy mutAted GeneS) are associated with late replication time. Diagnostic accuracy for high TMB group did not differ between TTN-TMB and TMB assessed by tNGS. Classification modeling by machine learning using TTN-TMB for MSI-H diagnosis was constructed, and the diagnostic accuracy was 0.873 by area under the curve in external validation. *TTN* mutation was enriched in samples possessing high immunostimulatory signatures. We suggest that the mutation load within TTN represents high TMB status.

## Introduction

Genomic instability is an important characteristic of cancers that results in an increased number of genetic alterations. In colorectal cancer (CRC), it has become apparent that genomic instability is related to tumorigenesis.^1,2^ CRCs can be divided into two distinctive subgroups, the hypermutated group and the non-hypermutated group, at the genomic level.^3^ Hypermutated tumors are characterized by an increased frequency of base substitutions, insertions, and deletions of one or several nucleotides. These nucleotide alterations are often related to defective base excision repair system components, including defects in DNA polymerase δ and ε (*POLD* and *POLE*).^4,5^ Additionally, defects in the DNA mismatch repair (MMR) system components, including *MSH2*, *MLH1*, *MSH6*, *PMS1*, and *PMS2*, are another major contributor to hypermutated tumors.^3^ A defect in the MMR system is characterized by an increase in random insertions or a reduction of the number of oligo-nucleotide repeats within microsatellite sequences, ultimately resulting in the microsatellite instability-high (MSI-H) phenotype.^1,6,7^ Therefore, tumors exhibiting a loss of these DNA repair systems are expected to possess an increased spontaneous mutation rate across the entire genome.

Recent studies have provided concrete evidence that hypermutated tumors respond better to immunotherapies.^8^ Based on this, identifying patients harboring hypermutated tumors is suggested for optimal treatment. Whole-exome sequencing (WES) or targeted next-generation sequencing (tNGS) using a cancer gene panel are utilized to assess the tumor mutation burden (TMB).^9,10^ WES to determine TMB is not, however, typically performed during routine clinical diagnosis, as it is not time-effective and the storage of WES data is resource-intensive. TMB estimation using tNGS also possesses limitations. First, the accurate prediction of TMB by WES requires a panel size of greater than 300 genes or 1 Mb,^9^ and second, the cut-off for high TMB varies depending on the targeted genes, the panel size, and the bioinformatics pipeline. Given this, standardized TMB measurement methods are needed.^11^

In this context, if single gene testing could predict TMB determined by WES or tNGS, then those limitations could be avoided. Thus, we examined if mutation status within a single gene could be representative of TMB as assessed by larger-scale sequencing such as WES or tNGS. For this approach, we analyzed if the mutation count in a single gene could reflect the TMB by WES (TMB-WES) using a more reliable pan-cancer cohort (*n* = 10,224) obtained from The Cancer Genome Atlas (TCGA) MC3 project.^12^ We then subsequently validated our findings using an independent dataset.

## Results

### Mutations within *TTN* represent TMB as assessed by WES in pan-cancer data

**Fig. 1:**
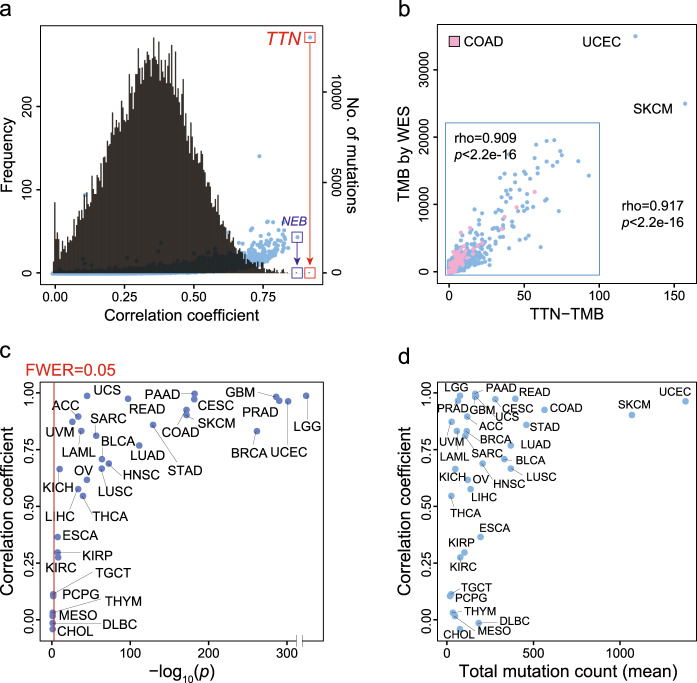
Pan-cancer data analysis. **a** Histogram (black line) of the correlation coefficients between the mutation number in each gene and the total mutation count in all genes across the pan-cancer cohort (Pearson correlation test). The scatter plot (blue dots) indicates mutation counts in 20,969 genes from all samples. **b** Correlation between mutation counts within the *TTN* gene (TTN-TMB) and TMB by WES (TMB-WES) for the pan-cancer cohort (*n* = 10,224) (Pearson correlation test). **c** Correlation coefficients between TTN-TMB and TMB-WES for 33 tumor types (Pearson correlation test). **d** Correlation coefficients of TTN-TMB and TMB-WES (*Y*-axis) and total mutation count (mean) (*X*-axis) for each tumor type. TMB tumor mutation burden, WES whole-exome sequencing.

Initially, 10,224 samples across 33 cancer types were used to assess if the mutation count within a single gene correlated with TMB as determined by WES. All candidate genes (*n* = 20,969) were evaluated to identify the gene that was most strongly correlated with TMB as assessed by WES. For each of the 20,969 genes, the correlation coefficient and the associated *p*-value between the number of somatic mutations in each gene and the total number of all somatic mutations were calculated. In this analysis, the mutation frequency within *TTN* was most strongly correlated with TMB-WES in the pan-cancer cohort (Fig. 1a). The correlation coefficient was 0.917 (*p* < 2.2e-16, Fig. 1b). When two samples exhibiting extremely high mutation rates were excluded, the correlation coefficient remained as high as 0.909 (*p* < 2.2e-16, Fig. 1b, inset box). Therefore, we selected *TTN* as the single gene for further detailed analysis.

Next, the correlation between the TTN-TMB and the TMB-WES was determined for each cancer type. TTN-TMB was significantly correlated with TMB-WES across all 33 tumor types, with the exception of PCPG, TGCT, THYM, MESO, DLBC, and CHOL (FWER, raw *p*-value*33, <0.05) (Fig. 1c**)**. Tumors with high TMB tend to show high correlation coefficients (Fig. 1d). Among these tumor types, COAD, CESC, SKCM, READ, UCEC, PRAD, and GBM exhibited the highest *TTN* mutation count, and this gene possessed the high correlation coefficient (>0.9) of all tested genes (Supplementary Fig. 1 and Supplementary Data 1). In STAD, BLCA, LUSC, LUAD, LIHC, HNSC, ACC, and LAML, *TTN* also showed the highest mutation count, although the correlation coefficient was relatively low (rho < 0.9, *p* < 0.05) among all tested genes. The most highly correlated genes for each tumor type are summarized in Supplementary Data 1. Additionally, the most highly mutated genes for each tumor type are summarized in Supplementary Data 2. These results suggest that TTN-TMB could represent TMB in the context of multiple tumor types.

### TTN-TMB and TMB-WES in TCGA CRCs

**Fig. 2:**
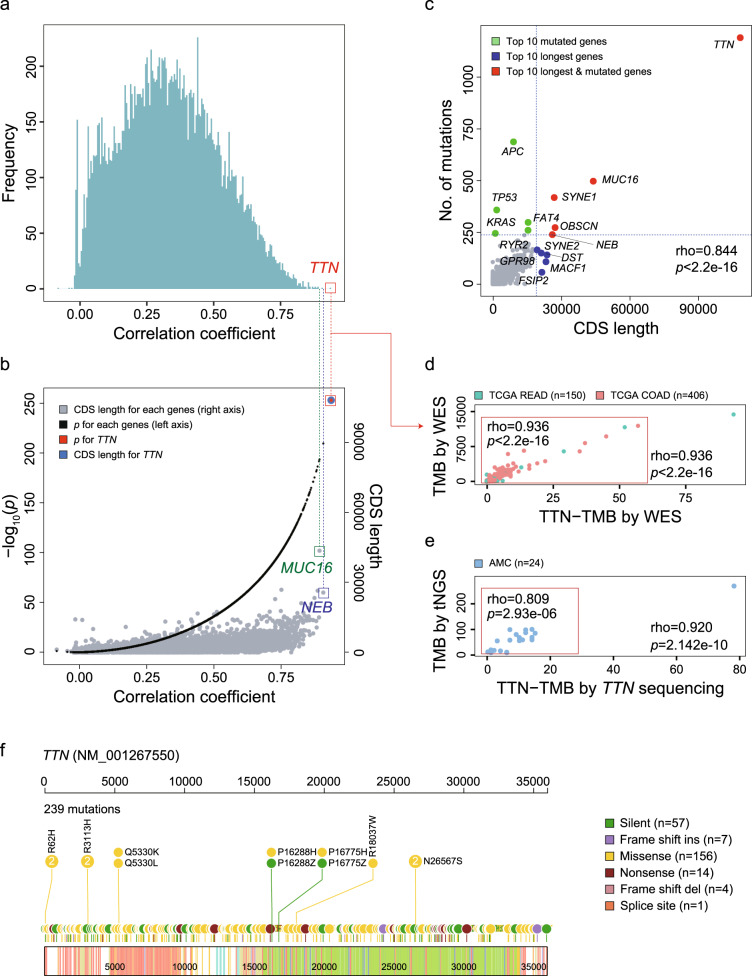
Correlation analysis of two independent colorectal cancer cohorts. **a** Histogram of the correlation coefficients between the mutation count of each gene and the total mutation count in a TCGA colorectal cancer (CRC) cohort (*n* = 556) (Pearson correlation test). **b** *p*-values (black dots) for the correlation coefficient and CDS lengths (gray dots) for each gene (Pearson correlation test). **c** Comparison of CDS lengths and mutation counts in all observed genes within the TCGA CRC cohort. Red dots indicate top genes possessing both high CDS lengths and frequent mutation, blue dots indicate genes with relatively large CDS lengths and infrequent mutation, and green dots indicate genes with relatively short CDS lengths and frequent mutation. Correlation of TTN-TMB, TMB-WES, and TMB-tNGS in the TCGA CRC cohort (**d**) and the AMC cohort (**e**) (Pearson correlation test). **f** Lollipop plot of all reported variants of the *TTN* gene in the AMC cohort. TMB tumor mutation burden, WES whole-exome sequencing, tNGS targeted next-generation sequencing.

TTN-TMB was subjected to further detailed analysis within the COAD (*n* = 406) and READ (*n* = 150) cohorts from TCGA. Of the 556 TCGA CRC cases, 75 (13.5%) were MSI-H, 477 (85.8%) were non-MSI, and MSI status was not available for the four remaining cases. Correlation coefficients and *p*-values for all 19,360 genes were calculated, and mutations within *TTN* exhibited the highest correlation (Fig. 2a and Supplementary Data 3) and the longest CDS length (Fig. 2b) among all tested genes. The gene possessing the second highest correlation coefficient was *NEB* (rho = 0.907, *p* = 5.65e-210, Fig. 2b). Additionally, the relationship between mutation count and CDS length in 19,360 genes was also evaluated, and the high total mutation number within the *TTN* gene was strongly related to its CDS length (Fig. 2c). We determined that the CDS length correlated with mutation count for each gene (rho = 0.844, *p* < 2.2e-16). As expected, however, mutation rates within cancer-associated genes, such as *APC, TP53, and KRAS*, were not associated with their CDS lengths (Fig. 2c). Correlation plots between TTN-TMB and TMB-WES for all CRC samples are shown in Fig. 2d (rho = 0.936, *p* < 2.2e-16), and the correlation coefficient was the same even when cases exhibiting extremely high mutation rates were excluded (Fig. 2d, inset).

### An additional factor other than gene length can explain frequent mutation in *TTN*

**Fig. 3:**
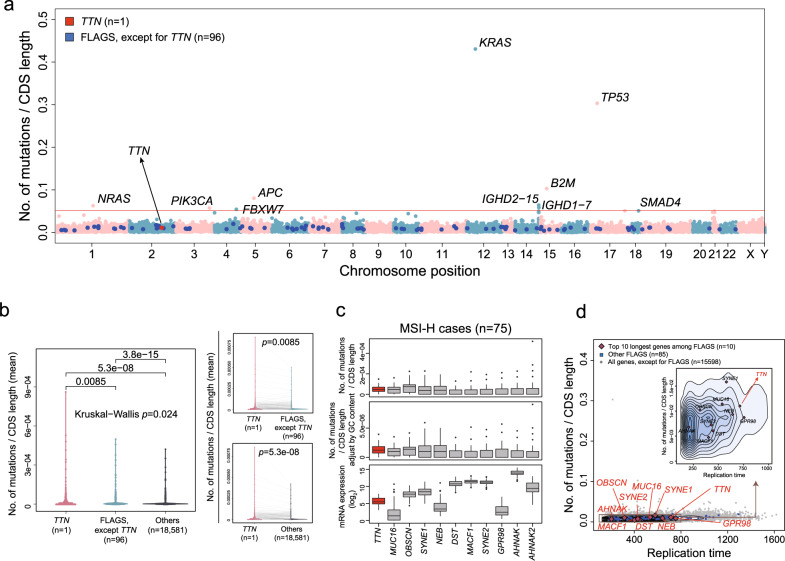
*TTN* mutation frequency and replication time. **a** Mutation frequency adjusted by coding sequence (CDS) length. **b** Mutation frequency adjusted by CDS among *TTN*, other FLAGS, and remaining 18581 genes (*p-*value by paired Wilcoxon rank-sum test). **c** Mutation frequency adjusted by CDS length and by both CDS length and GC content, and mRNA expression level of the top 11 FLAGS, including *TTN* in CDS length in the MSI-H group. **d** Association between mutation frequency and replication time in FLAGS, including *TTN*.

We found that the *TTN* mutation rate is higher than the expected mutation rate of other FLAGS (FrequentLy mutAted GeneS),^13^ which are known primarily as non-driver passenger genes that can be used to show frequent mutation in cancer. Additionally, frequent mutations in these genes are usually associated with a long gene length. The mutation count was normalized according to CDS length (Mutation count/CDS length) for each gene (Fig. 3a). The mutation rate normalized according to CDS length was significantly higher in *TTN* than in other FLAGS or remaining genes (Fig. 3b). A similar pattern was observed even when the mutation rate was normalized by GC content (%) in addition to CDS length (Fig. 3c), and gene expression levels varied for these FLAGS that possessed long CDS length (Fig. 3c). We suggest that one possible explanation for these observations may be the later replication time of *TTN* compared to that of other FLAGS possessing long CDS length (Fig. 3d), as mutation frequency is correlated with DNA replication timing.^14^ This finding has been also previously described by Tan et al.^15^

### Mutation characteristics according to *TTN* gene sequencing and targeted sequencing within the AMC cohort

**Fig. 4:**
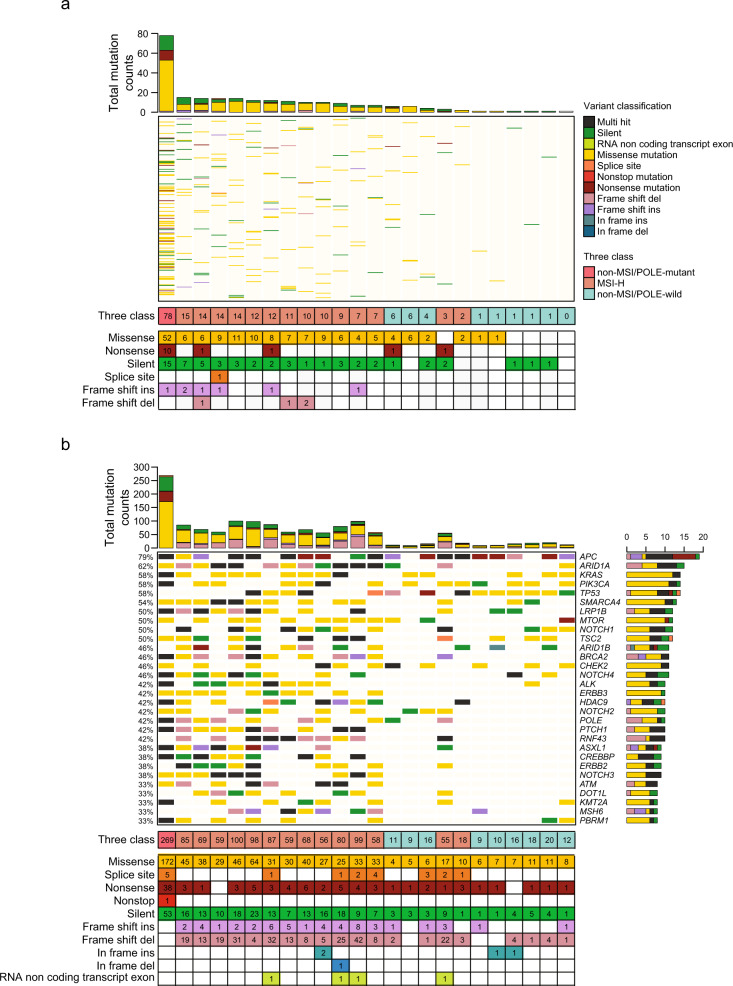
Mutational profiles of the AMC cohort. **a** Mutation profiles determined by *TTN* sequencing according to MSI and *POLE* mutation status. **b** Mutation profiles determined by targeted next-generation sequencing in the same samples.

Next, we performed *TTN* gene sequencing of 24 CRCs in which targeted NGS data were available to validate the findings from the pan-cancer cohort. For the AMC cohort (*n* = 24 CRCs), 14 patients were MSI-H, and the remaining 10 were MSS, including one patient with a *POLE* mutation. The median ages at diagnosis within the MSI-H and MSS groups were 51.5 years (range, 21–80) and 58 years (range, 45–69), respectively. Of the 14 MSI-H CRCs, three (21.43%) were mucinous adenocarcinomas, and the remaining 11 (78.57%) were conventional adenocarcinomas. All 10 CRC cases exhibiting MSS (100%) were histologically classified as conventional adenocarcinoma. Patient characteristics and associated mutational features for MMR-related genes and the *POLE* gene are summarized in Supplementary Table 1. In the *TTN* panel assay, there were 239 somatic alterations within non-intronic regions, including 156 missense mutations (65.27%), 57 silent mutations (23.85%), 14 nonsense mutations (5.86%), one splice site mutation (0.42%), seven frame shift insertions (2.93%), and four frame shift deletions (1.67%) (Fig. 4a). The landscape of *TTN* mutations in the 24 samples, along with their MSI and *POLE* mutation status, is shown in Fig. 4a. InDel mutations were not found in the non-MSI/*POLE*-wild-type group. One *POLE*-mutated case exhibited ultra-mutation within the *TTN* gene. For targeted NGS using the OP_AMCv3 assay, there were 1381 somatic alterations in the protein coding region, including 705 missense mutations (51.05%), 257 frame shift deletions (18.61%), 251 silent mutations (18.18%), 90 nonsense mutations (6.52%), 49 frame shift insertions (3.55%), 19 splice sites (1.38%), four in-frame insertions (0.29%), four RNA non-coding transcript exon variants (0.29%), one in-frame deletion (0.07%), and one non-stop mutation (0.072%). The mutational landscape of the 30 most frequently mutated genes is provided in Fig. 4b.

### Correlation between TTN-TMB and TMB-tNGS

We evaluated if TTN-TMB correlated with targeted sequencing using the 24 CRCs of the AMC cohort. *TTN* somatic mutation counts were also significantly correlated with the TMB based on targeted sequencing (TMB-tNGS) (Fig. 2e, rho = 0.920, *p* = 2.142e-10). When one sample exhibiting extremely high mutation rates were excluded, the correlation coefficient was 0.809 (*p* = 2.93e-06, Fig. 2e, inset). The mutation pattern of *TTN* was characterized by spontaneous mutations throughout all exons of *TTN*, and no hotspots were found (Fig. 2f). These findings suggest that the somatic mutation frequency of *TTN* could be representative of TMB determined by targeted sequencing and by WES.

### Comparison of TTN-TMB within genomic instability groups

**Fig. 5:**
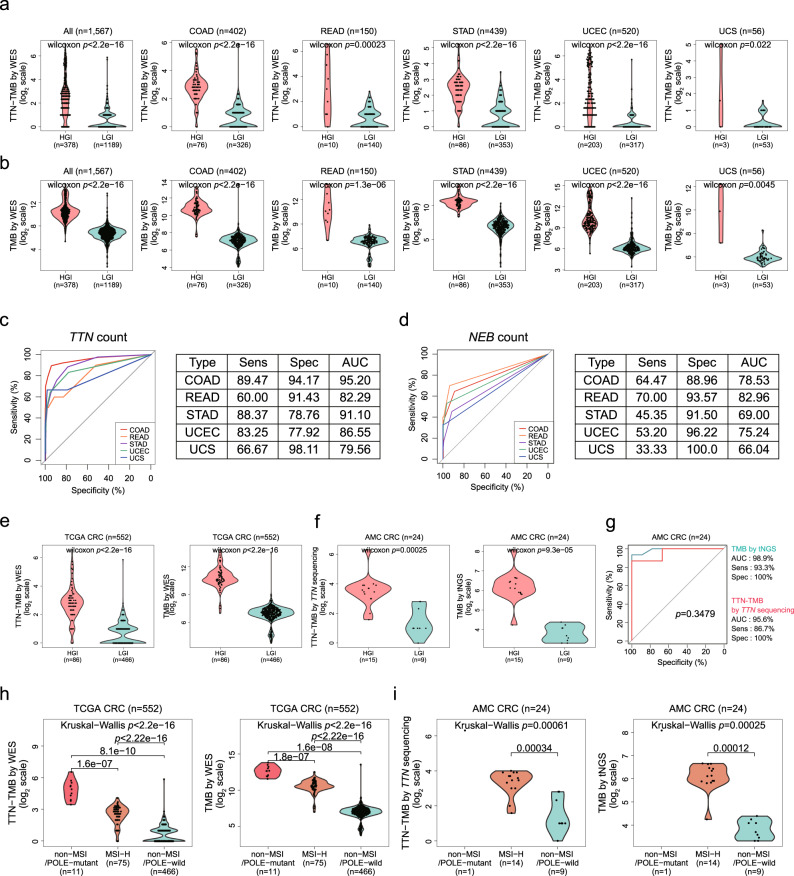
Comparison of TTN-TMB according to MSI and *POLE* mutation status. Mutation counts within the *TTN* gene (**a**) and total mutation counts of all genes (**b**) according to HGI and LGI samples in the TCGA cohort (*n* = 1567, Wilcoxon rank-sum test) for each tumor type. Diagnostic performance of *TTN* (**c**) and *NEB* (**d**) for HGI in each tumor type. The differences in *TTN* mutation count between the HGI and the LGI groups in the TCGA CRC cohort (**e**) and the AMC cohort (**f**) (Wilcoxon rank-sum test). **g** No differences in diagnostic accuracy for HGI and LGI were observed between TTN-TMB and TMB by targeted NGS (tNGS) in the AMC cohort. The differences in *TTN* mutation count among the *POLE*-mutated group, the MSI-H group, and the remaining group in the TCGA CRC cohort (**h**) and the AMC cohort (**i**) (Wilcoxon rank-sum test or Kruskal-Wallis test). HGI high genomic instability, LGI low genomic instability, TMB tumor mutation burden, WES whole-exome sequencing, NGS next-generation sequencing, AUC area under the curve.

We evaluated if TTN-TMB could be used to discriminate between HGI and LGI in COAD (*n* = 402), READ (*n* = 150), STAD (*n* = 439), UCEC (*n* = 520), and UCS (*n* = 56), as these tumor types possess MSI-H status information. Of the 1567 patients from the TCGA pan-cancer cohort, the significant differences in TMB measured by TTN-TMB (*p* < 2.2e-16, Fig. 5a) were found between HGI and LGI, likely as measured by TMB-WES (*p* < 2.2e-16, Fig. 5b). In each tumor type, we tested the diagnostic performance using mutation counts within *TTN*. The diagnostic performance of *TTN* was high, and the COAD exhibited the highest AUC value (95.2) (Fig. 5c), which was much better than that obtained using *NEB*, which yielded the second highest correlation coefficient (Fig. 5d). When COAD and READ were combined into CRC, *TTN*-TMB (*p* < 2.2e-16) and TMB-WES (*p* < 2.2e-16) yielded also both significantly differences between HGI and LGI tumors (Fig. 5e), and these relationships were also observed within the AMC cohort (*p* = 0.00025, Fig. 5f left for TTN-TMB; *p* = 9.3e-05, Fig. 5f right for TMB-tNGS). The mean mutation numbers within *TTN*-based on *TTN* sequencing in HGI and LGI tumors were 14.53 ± 17.99 and 2.33 ± 2.35, respectively. TTN-TMB yielded no significant differences compared to TMB-tNGS in regard to diagnostic performance in the classification of HGI and LGI (AUC, 0.956 vs. 0.989, *p* = 0.3479, Fig. 5g). When the cases within the TCGA were classified into three groups (non-MSI/*POLE*-mutant, MSI-H, and non-MSI/*POLE*-wild-type), TTN-TMB and TMB-WES were also both significantly different among the three groups (TTN-TMB: *p* < 2.2e-16, Fig. 5h, left; TMB-WES: *p* < 2.2e-16, Fig. 5h, right). These findings were also repeated in the AMC cohort (TTN-TMB: *p* = 0.00061, Fig. 5i left; TMB-tNGS: *p* = 0.00025, Fig. 5i right).

### Classification model construction using TTN-TMB for MSI-H and external validation

**Fig. 6:**
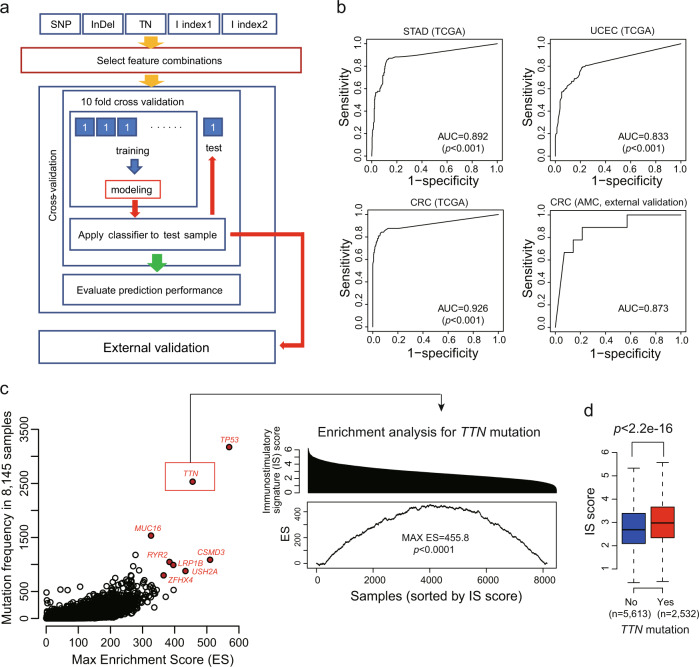
Prediction model construction using TTN-TMB and association between immunostimulatory signature and mutation. **a** Overall process of constructing the prediction model to classify MSI-H and MSS by machine learning. **b** Internal and external validation of the diagnostic model for MSI-H constructed using Random Forest machine learning in STAD, UCEC, and CRC. **c** Enrichment scores associated with immunostimulatory signature (IS) for each gene mutation. *TTN* mutations were significantly enriched in samples possessing high IS score (permutated *p* < 0.0001; 10,000 random sample class permutations). **d** IS score was significantly higher in the *TTN* mutated group than in the *TTN* wild-type group (*p* < 2.2e-16, Wilcoxon rank-sum test). AUC area under the curve, N total mutation number.

Next, we built a prediction model using a Random Forest machine learning (Fig. 6a) to classify MSI-H and MSS based on TTN-TMB for CRC, STAD, and UCEC. We used a Random Forest Model, as this is one of the most robust prediction models and MSI-H frequencies are different between the training (TCGA) set and the validation (AMC) set. Patient information, including age and sex, was not selected during the model construction. In prediction performance, AUC was 0.892 (*p* < 0.001), 0.833 (*p* < 0.001), and 0.926 (*p* < 0.001) for STAD, UCEC, and CRC, respectively. Among these, CRC exhibited the highest predictive model performance, and the model was applied to 23 AMC CRCs for external validation. The AUC was 0.873 (Fig. 6b), despite the use of different mutation calling methods between the TCGA and AMC sets. These findings suggest that TTN-TMB can be used as a diagnostic marker for MSI-H specifically in CRC.

### Immunostimulatory signature enriched in tumors with TTN mutation

We evaluated the existence of any relationship between *TTN* mutation and immunostimulatory signature using 8145 pan-cancer data sets that contained both mutation and gene expression data for enrichment analysis. In this analysis, we identified *TP53* mutation has the highest max ES (score = 569.5) for immunostimulatory signature (Fig. 6c), indicating that *TP53* mutation was more frequently found in tumors with high immunostimulatory signature. *TTN* mutation was also ranked within the top 5 in max ES (score = 455.8, permutated *p* < 0.0001, Fig. 6c), and the IS score was significantly high in the *TTN* mutated group (*p* < 2.2e-16) compared to that of the *TTN* non-mutated group (*p* < 2.2e-16, Fig. 6d), suggesting that *TTN* mutation is directly or indirectly associated with immunogenic tumors.

## Discussion

Through computational analyses of 33 cancer types from pan-cancer databases and the AMC cohort, we demonstrated that the mutation count within the *TTN* gene can represent the TMB status within various tumor types and can be used to predict the MSI-H status specifically in CRC. A high correlation was typically observed in the tumors known as hypermutated tumor types, such as SKCM, CRC, UCEC, LUAD, BLCA, that frequently possess more than 10 mutations per Mb.^21,30^ However, TTN-TMB revealed low correlation coefficients in tumor types that possessed low mutation rates, including breast, kidney, and thyroid tumors. Therefore, TTN-TMB may possess limitations in regard to those tumor types.

We acknowledge that current gene panel testing utilizing NGS can provide actionable gene mutations and can identify TMB, and therefore, a *TTN*-based sequencing assay would provide no useful information beyond TMB. However, in regard to the current gene panel testing using NGS, it is known that the panel size is recommended to be greater than 1 MB to detect the TMB with a narrow range of confidence interval.^16,17^ Additionally, actionable mutations linked to approved target agents are limited for several genes according to tumor type. Therefore, gene panel analysis may be more efficient when using *TTN* in combination with a small number of targetable genes according to tumor type. Based on this, panel sizes could be reduced to a smaller number of genes. When the number of tested genes is reduced, there are several advantages that include reduced review time for variant interpretation, faster computational analysis, efficient DNA library construction (clinical NGS testing uses formalin-fixed paraffin-embedded tissue, and DNA within these tissues undergoes severe degradation that results in capture difficulties that make it difficult to obtain high read depth evenly from the multiple genes located at different chromosomal location, ultimately leading to false negatives in variant calling at low read depth location), and faster sequencing time due to reduced library size (Supplementary Table 2). Additionally, a larger number of assays can be processed simultaneously on a given NGS platform due to reduced library size.

We used computer-based modeling to randomly generate 100,000 sets composed of 2–25 genes to determine whether sets of a similar size to *TTN* can provide similar power for detecting TMB or MSI-H (Supplementary Fig. 2). This analysis suggests that optimal gene sets that possess a similar size to *TTN* may provide similar power for detecting TMB or MSI-H, and increased panel size results in smaller confidence intervals.^16,17^ However, this analysis also indicate that multiple gene combinations are required to obtain similar performance to that of the individual *TTN*. Determining the optimal gene set to satisfy all conditions, including the highest mutation rate and the highest correlation coefficient or accuracy, may not, however, be easy and may be dependent upon tumor types. In this study, we would like to emphasize that a single gene, although large, can effectively represent the TMB in a manner similar to that of multiple gene combinations. This method may also be useful under specific conditions such as those encountered in a clinical setting using the Amplicon-based hotspot panel, as it is difficult to define the TMB using NGS of circulating tumor DNA (ctDNA). Evaluation of TMB by analyzing ctDNA is becoming a promising diagnostic method for the selection of immunotherapy candidates, and NGS cancer panels specific for ctDNA inevitably currently possess considerable panel size limitations.

Initially, most studies employed WES for estimating TMB, and they demonstrated that a high TMB was associated with a higher likelihood of response to immunotherapy. This approach has not, however, been successfully applied to clinical testing due to the high cost and turn-around time. For this reason, TMB using targeted NGS has been assessed, and Rizvi et al. demonstrated that TMB estimated using targeted gene panels was highly correlated with TMB assessed by WES.^18^ Campesato et al., however, demonstrated that gene panels that included fewer than 150 genes are less accurate in their estimation of TMB by WES than are larger panels.^19^ Additionally, Buchhalter et al.^16^ demonstrated that the optimal panel size (between 1.5 and 3 Mb) is an essential factor for the precise estimation of TMB. However Lyu et al.^20^ revealed that a small gene set could be used for estimating TMB.

Although we sequentially validated the diagnostic utility of the *TTN* gene mutation count in CRC, our discovery analyses indicated that *TTN* gene mutation count may also be useful for prediction of TMB in other tumor types. The strong diagnostic value of the *TTN* gene mutation count could be attributable to the low biological selection pressure of *TTN* functional loss. As the protein coding regions of biologically important genes are subject to selection pressure, mutations affecting those regions are positively or negatively selected, and they may be observed commonly across tumors having a different TMB. In contrast, genes that are relatively less important for cell proliferation or survival are less susceptible to selective pressure, and thus, mutations affecting those genes are able to accumulate, ultimately contributing significantly to TMB. In this sense, the *TTN* mutation count may provide a good candidate for a surrogate for TMB status.

In this study, we demonstrated that mutation count in a single gene, *TTN*, can be used to estimate TMB. To our knowledge, this is the first approach to demonstrate the relationship between somatic mutations in a single gene and the TMB determined by targeted NGS or WES. Additionally, our results also indicate that the *TTN* mutation profile could be used as a predictor of MSI-H, at least in regard to CRC. Incorporating *TTN* into a given gene panel design may increase the efficiency for the detection of TMB.

## Methods

### Study design

This retrospective study was designed to identify a single gene that could represent TMB as assessed by WES or targeted NGS. The overall study design is summarized in Supplementary Fig. 3. A correlation analysis was performed using 10,224 samples across 33 cancer types from TCGA. Additionally, among this pan-cancer cohort, COAD (*n* = 402 of 406; four cases have no MSI information), READ (*n* = 150), STAD (*n* = 439), UCEC (*n* = 520 of 530; 10 cases have no MSI information), and UCS (*n* = 56 of 57; one case has no MSI information) with MSI status information were further analyzed for diagnostic accuracy of TTN-TMB to *POLE* mutation or MSI-H status. Additionally, external validation was performed for a CRC cohort, as this tumor type is a representative tumor type that is MSI-H. For external validation, tNGS and *TTN* gene sequencing analyses were performed on 24 CRCs from the Asan Bio-Resource Center at Asan Medical Center, Seoul, Korea (AMC cohort).

Finally, we assessed if TTN-TMB could classify tumors into subgroups according to MSI status and *POLE* mutation status (non-MSI/*POLE*-mutant, non-MSI/*POLE*-wild, and MSI-H). Of these three subgroups, the two that are categorized by a high mutation rate (MSI-H or non-MSI/*POLE*-mutant) were grouped together as the high genomic instability (HGI) group for analysis, and the remaining subgroup (non-MSI/*POLE*-wild-type) was considered the low genomic instability (LGI) group. This study was approved by the Institutional Review Board of Asan Medical Center. The institutional review board waived the requirement to obtain informed consent because all tested samples in this study were obtained from the Bio-Resource Center at Asan Medical Center.

### Pan-cancer cohort data used in this study

We used TCGA MC3 MAF v3 when examining somatic mutations (mc3.v0.2.8.PUBLIC.maf).^12^ The corresponding clinical data were obtained from the TCGA GDC portal. The dataset included the following cancer types: adrenocortical carcinoma (ACC, *n* = 92), bladder urothelial carcinoma (BLCA, *n* = 411), breast invasive carcinoma (BRCA, *n* = 1020), cervical and endocervical carcinoma (CESC, *n* = 289), cholangiocarcinoma (CHOL, *n* = 36), colon adenocarcinoma (COAD, *n* = 406), lymphoid neoplasm diffuse large B-cell lymphoma (DLBC, *n* = 37), esophageal carcinoma (ESCA, *n* = 184), glioblastoma (GBM, *n* = 393), head and neck squamous cell carcinoma (HNSC, *n* = 507), kidney chromophobe carcinoma (KICH, *n* = 66), kidney renal clear cell carcinoma (KIRC, *n* = 369), kidney renal papillary cell carcinoma (KIRP, *n* = 281), acute myeloid leukemia (LAML, *n* = 141), brain lower grade glioma (LGG, *n* = 512), liver hepatocellular carcinoma (LIHC, *n* = 363), lung adenocarcinoma (LUAD, *n* = 567), lung squamous cell carcinoma (LUSC, *n* = 485), mesothelioma (MESO, *n* = 82), ovarian serous cystadenocarcinoma (OV, *n* = 412), pancreatic adenocarcinoma (PAAD, *n* = 177), pheochromocytoma and paraganglioma (PCPG, *n* = 179), prostate adenocarcinoma (PRAD, *n* = 497), rectum adenocarcinoma (READ, *n* = 150), sarcoma (SARC, *n* = 236), skin cutaneous melanoma (SKCM, *n* = 466), stomach adenocarcinoma (STAD, *n* = 439), testicular germ cell tumors (TGCT, *n* = 145), thyroid carcinoma (THCA, *n* = 492), thymoma (THYM, *n* = 123), uterine corpus endometrial carcinoma (UCEC, *n* = 530), uterine carcinosarcoma (UCS, *n* = 57), and uveal melanoma (UVM, *n* = 80). Of these 33 tumor types, COAD and READ were merged into CRC. For the 33 cancer types, the number of samples is depicted in Supplementary Table 3.

### Patients and tumor specimens of the AMC cohort

For the validation cohort, 24 patients with CRC were selected from our previous study^21^ according to the following criteria: (1) tumors were evaluated using PCR-based analysis of microsatellite loci and (2) tNGS data were available. Of the 24 CRCs, 14 (58.3%) were the MSI-H phenotype, as confirmed by MSI PCR analysis, and the remaining 10 (41.7%) were microsatellite stable (MSS), with a *POLE* pathogenic mutation in one case. In this study, we attempted to balance the frequency of MSI (MSI-H vs. MSS = 1:1), as the aim of this study was to determine if *TTN* correlates with TMB.

### Microsatellite loci testing by PCR

MSI status in the AMC cohort (*n* = 24) was evaluated by PCR. Fluorescently labeled primers were used to amplify five different microsatellite loci, including two mononucleotide repeats (BAT-25 and BAT-26) and three dinucleotide repeats (D5S346, D2S123, and D17S250) in tumors and matched normal samples. MSI status was determined based on a different length of the PCR product within the tumor sample compared to that of the paired normal sample. Samples with instability in two or more of the five loci were defined as MSI-H. Samples with instability in one of the five loci were defined as microsatellite instability-low (MSI-L). Samples with no instability were defined as MSS. In this study, MSI-L and MSS were classified as non-MSI.

### Targeted NGS in the AMC cohort

BAM files from the tNGS for the AMC cohort (*n* = 24 CRCs) were obtained from our previous study.^21^ Detailed information on the targeted sequencing method has been described previously.^21^ Briefly, the targeted NGS panel, OncoPanel AMC version 3 (OP_AMCv3), was designed at AMC using SureDesign (Agilent Technologies, USA) and Genome Reference Consortium Human Build 37 (GRCh37) as the reference genome. This panel is ~1 Mb in size and contains 33,524 probes targeting a total of 382 genes, including the entire exons of 199 genes, 184 hotspots, and partial introns of eight genes often rearranged in cancer. The *TTN* gene was not included in this panel.

### *TTN* gene sequencing and data processing

For the AMC cohort (24 CRCs) with tNGS data, a *TTN* gene panel (Celemics, Korea) was designed to cover only the exonic regions of the *TTN* gene. Genomic DNA was extracted from formalin-fixed, paraffin-embedded (FFPE) tumor tissue, and a DNA library was prepared using the SureSelect XT custom kit (Agilent Technology) after DNA quality assurance. Pooled libraries were sequenced using an Illumina MiSeq (Illumina, USA). Sequencing was performed in tumor tissue without matched normal tissue. Sequenced reads were mapped to the GRCh37 using the Burrows-Wheeler Aligner (BWA) version 0.7.15^22^ under the default settings. PCR duplicate reads were identified and removed using MarkDuplicate of the Genome Analysis Tool Kit (GATK) version 4.0.2.1.^23^ Recalibration of the base quality was performed using ApplyBQSR of GATK version 4.0.2.1.^23^

### Variant calling and filtering to identify somatic variant candidates

Somatic variant candidate calling for single-nucleotide variant (SNV) and insertion/deletion mutation (InDel) was performed using the BAM file using Mutect2 in the tumor-only mode of GATK 4.0.2.1.^23^ The raw variants called by Mutect2 were additionally filtered out as follows. First, the raw variants generated using Mutect2^23^ were filtered out with the exception of “PASS” and “germline risk.” Second, the remaining variants were further filtered out using the following databases: (1) the Korean Reference Genome Database (KRGDB, http://coda.nih.go.kr/coda/KRGDB) and (2) an in-house panel of normals (PON). The remaining candidates were also filtered out using in-house criteria (total depth < 30 and variant read depth for SNV < 3 or variant read depth for InDel < 5). These somatic candidates were annotated using Variant Effect Predictor (VEP) version 91^24^ and converted to MAF format using vcf2maf version 1.6.15 (https://github.com/mskcc/vcf2maf). Then, the annotated candidates within intronic regions were removed. Next, as variants annotated as “germline risk” are included in the Genomic Aggregation Database (gnomAD) and because some of these variants may be somatic mutations, the variants considered to be true germline variants were filtered out based on the distribution of the “PASS” variants using a kernel density algorithm in the subsequent filtering step as follows:

(i) “PASS” variants were assumed to be true somatic mutations.
(ii) Distribution of the variant allele fraction of the somatic mutations was assumed to be nonparametric.

1 $$\begin{array}{*{20}{c}} {{\mathrm{Kernel}}\,{\mathrm{density}},f\left( x \right) = \frac{1}{{nh}}\mathop {\sum }\limits_{i = 1}^n K\left( {\frac{{x - xi}}{h}} \right)} \end{array}$$

x_i_ = variant allele fraction of each mutation in passed somatic mutations;

2 $$\begin{array}{*{20}{c}} {K\left( u \right) = \frac{1}{{\sqrt {2\pi } }}e^{ - \frac{1}{2}u^2};h = \left( {\frac{{4\sigma ^5}}{{3n}}} \right)^{\frac{1}{5}} \approx 1.06\sigma n^{ - \frac{1}{5}}} \end{array}$$

If *f*(*x*) [for variant_i_ in germline risk] ≈ 0, the variant was discarded.

Among the filtered variants in this step, the variants were rescued if they fulfilled these two criteria: (1) predicted to be deleterious variants in Sorting Intolerance From Tolerant (SIFT),^25^ and (2) predicted to be probably or possibly pathogenic variants in Polymorphism Phenotyping version 2 (PolyPhen-2).^26^ For *POLE*, only mutations within the exonuclease domain were considered pathogenic. In this study, the final remaining candidates were considered somatic mutations.

### Estimation of tumor mutation burden (TMB)

For the AMC and TCGA cohort, all SNV and InDel types, including synonymous and non-synonymous mutations in all exonic regions and splice sites, were used to calculate TMB,^16^ and the same methods were applied for all data used in this study. The TMBs measured by tNGS and WES were defined as the TMB-tNGS and TMB-WES, respectively. The total mutation count within the *TTN* gene was defined as the TTN-TMB. For all annotated genes harboring mutations (*n* = 19,360) in the TCGA CRC cohort, coding sequence (CDS) lengths were obtained from the Ensembl BioMart database.^27^

### Correlation analysis

Pearson correlation analyses between the total mutation count detected by NGS and the mutation count in each gene were performed for all cases, and correlation coefficients (rho) and *p*-values were obtained for each gene. The correlation analysis is described in detail in Supplementary Fig. 4.

### Immune stimulatory signature score based on the gene expression

We downloaded normalized gene expression data for 8145 cases with DNA sequencing data from Broad GDAC Firehose (https://gdac.broadinstitute.org/). Briefly, these data were upper-quartile normalized gene expression data generated from RNA sequencing. The data included the expression levels for a total of 20,502 genes. The gene expression level was further transformed by log2(expression value + 1). We selected known genes, including *IFNG*, *IL2*, *IL12A*, *IL12B*, *IL15*, and *TNF*, that were related to immunostimulatory signals (https://www.qiagen.com/ie/shop/pcr/primer-sets/rt2-profiler-pcr-arrays/?catno=PAHS-181Z#geneglobe). The immunostimulatory signature (IS) score was then simply defined as the average of the expression values of these six genes from the log2 transformed dataset.

### DNA replication time

DNA replication times for 17,667 genes provided by previous studies^14,28^ were used. The replication time indicates the order in which segments of chromosomal DNA duplicated at a particular time during the S-phase.^29^ A lower replication time indicates an earlier replication.

### Enrichment analysis of *TTN* mutation in the immune stimulatory signature

We used an enrichment score^30,31^ to determine if cases with *TTN* mutation were enriched among cases with high immune stimulatory signature across the whole sample. Briefly, a total of 8145 pan-cancer cases with corresponding RNA sequencing expression data were decreasingly ordered by immune stimulatory signature score. We then calculated the enrichment score, which was normalized by Kolmogorov-Smirnov statistics,^30,31^ as follows.

3 $$\begin{array}{l}{\mathrm{Enrichment}}\,{\mathrm{score}}\,\left( {\mathrm{ES}} \right)\,{\mathrm{for}}\,TTN \\= {{\mathrm{MAX}}\left( {{\sum \limits_{j = 1}^n} \sqrt {\frac{{N - G}}{G}}\, \left( {\mathrm{if}}\,{TTN}\,\,{\mathrm{in}}\,{\mathrm{Ptj}} \right) - {\sum \limits_{j = 1}^n} \sqrt {\frac{G}{{N - G}}}\, \left( {\mathrm{if}}\,{TTN}\,\,{\mathrm{not}}\,{\mathrm{in}}\,{\mathrm{Ptj}} \right)} \right)}\end{array}$$

*N* = total patient (Pt) number; G = number of patients with *TTN* mutation.

The ES reached a higher positive score when samples within the *TTN* mutated group were consistently ranked at the top of the sample list. The maximum ES was obtained when the N samples in the *TTN* mutated group were ranked as the top N most mutated samples among the 8145 samples. We permuted the *TTN* class label 10,000 times, and each time, we recorded the maximum ES generating background distribution. The permutated *p*-value was then calculated by

4 $$\begin{array}{*{20}{c}} {\tilde p = B^{ - 1}\mathop {\sum}\limits_{b = 1}^B I \left( {{\mathrm{maxES}_{0}} \le {\mathrm{maxES}_{\rm b}}} \right),B = 10,000} \end{array}$$

### Data visualization

MAF files for the AMC cohort were visualized using R package maftools version 1.7.05.^32^ A lollipop plot was generated using PCGP protein paint (https://pecan.stjude.cloud/proteinpaint).

### Classification modeling using machine learning with internal and external validation

To test the diagnostic accuracy of TTN-TMB, a classification model was constructed using the SNV count within *TTN*, the InDel count within *TTN*, the total mutation number (SNV + InDel) within *TTN*, index 1 (InDel/[SNV + InDel]), and index 2 (InDel/SNV). To construct the model, a Random Forest model was used, and the model was constructed to predict MSI-H versus MSS as a binary outcome for 541 CRCs, 438 STADs, and 478 UCECs. The UCS cohort was excluded due to only 2 MSI-H cases. To evaluate the predictive performance of the prediction model, a 10-fold cross-validation (CV) procedure was used as follows:

Step 1—The total data were randomly divided into 10 equally sized subsets.

Step 2—A single subset was used as the validation data, and the remaining nine subsets were used as training data.

Step 3—Random Forest was applied to the training set to fit a prediction model.

Step 4—A fitted prediction model was applied to the validation data, and the predicted probabilities were calculated.

Step 5—Steps 2–4 were repeated 10 times.

Step 6—After the cross-validation was completed, the predicted probability values of all samples calculated by 10-fold CV were combined together. A single ROC curve was drawn according to Simon et al.,^33^ and the area under the curve (AUC) value was calculated. To remove the overfitting bias of 10-fold CV as detailed by Simon et al.^33^, we calculated a permutation *p*-value from 10,000 random permutations as follows; (1) compute naive AUC value (*AUC*_0_) from the 10-fold CV procedure for the original data, (2) compute AUC value (*AUC*_b_) from the 10-fold CV procedure for the b-*th* permuted data (b = 1,…,B), (3) calculate a permutation *p*-value by

5 $$\begin{array}{*{20}{c}} {\tilde p = B^{ - 1}\mathop {\sum}\limits_{b = 1}^B I \left( {{\mathrm{AUC}_{0}} \le {\mathrm{AUC}_{\rm b}}} \right),B = 10,000} \end{array}$$

An external validation using the AMC cohort was performed for the CRC type by creating a prediction model with total CRC TCGA data.

### Statistical analysis

Wilcoxon rank-sum tests or Kruskal-Wallis tests were performed to compare the differences in continuous variables, including TMB. All reported *p*-values are two-sided, and *p* < 0.05 was considered statistically significant. All statistical analyses were performed using R version 3.5.2.

### Reporting summary

Further information on research design is available in the Nature Research Reporting Summary linked to this article.

## Supplementary information

Supplementary Information

Supplementary Data 1

Supplementary Data 2

Supplementary Data 3

Reporting Summary

## Data Availability

The TCGA data used in this study derived from public domain resources (https://gdac.broadinstitute.org and www.synapse.org/#!Synapse:syn7214402/files) were freely available. For the AMC cohort (*n* = 24), raw *TTN* gene-targeted sequencing data have been deposited in the European Genome-Phenome Archive (EGA) repository under accession number (EGAS00001004009).
