# Supplementary material for: Spontaneous mutations in the single TTN gene represent high tumor mutation burden
Source: NPJ Genom Med. 2020 Jan 14;5:33. doi: 10.1038/s41525-019-0107-6 (PMC7424531; doi:10.1038/s41525-019-0107-6)
Supplement: Supplementary file 1 — Supplementary Information [file 41525_2019_107_MOESM1_ESM.pdf]

Supplementary Figure 1

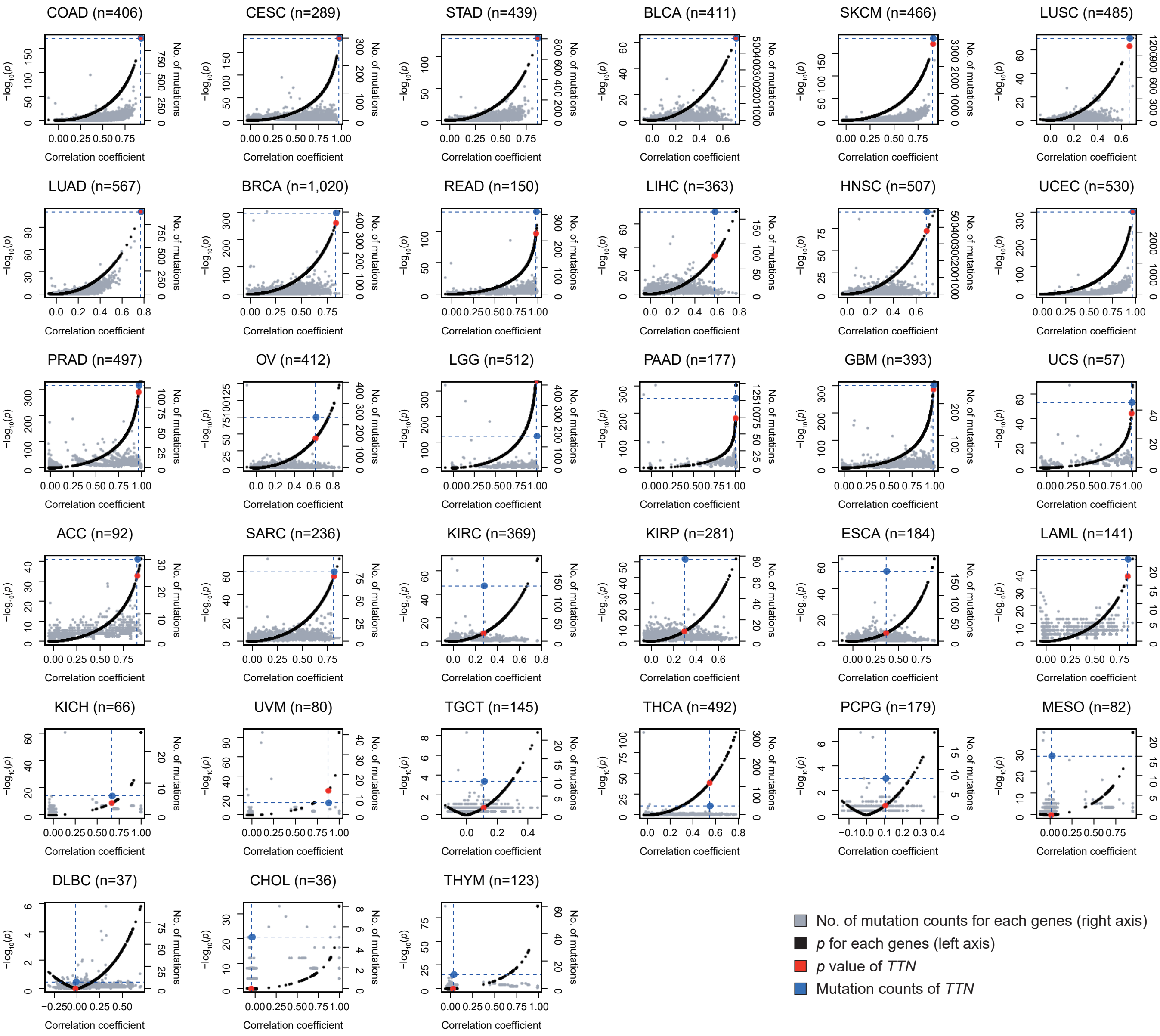

Supplementary Figure 1. Distribution of correlation coefficients and mutation counts for all tested genes within 33 tumor types. For each tumor type, correlation coefficients and *p*-values between all observed genes and total mutation counts are shown (left axis for *p*-values, red dot: *TTN*, black dot: others) (right axis for mutation number, blue dot: *TTN*, grey dot: others).

Supplementary Figure 2

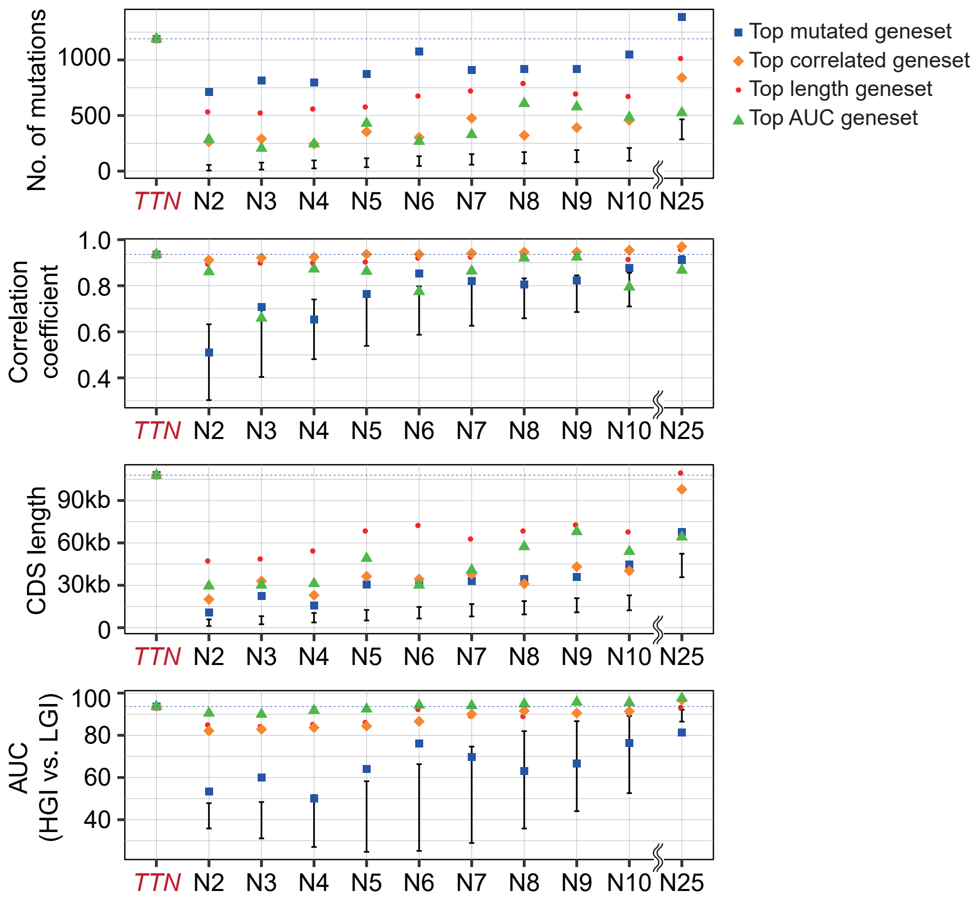

Supplementary Figure 2. Simulations using random combinations of multiple genes (100,000 times for each set) in comparison to TTN-TMB. N* indicates the number of genes in a given combination.

Supplementary Figure 3

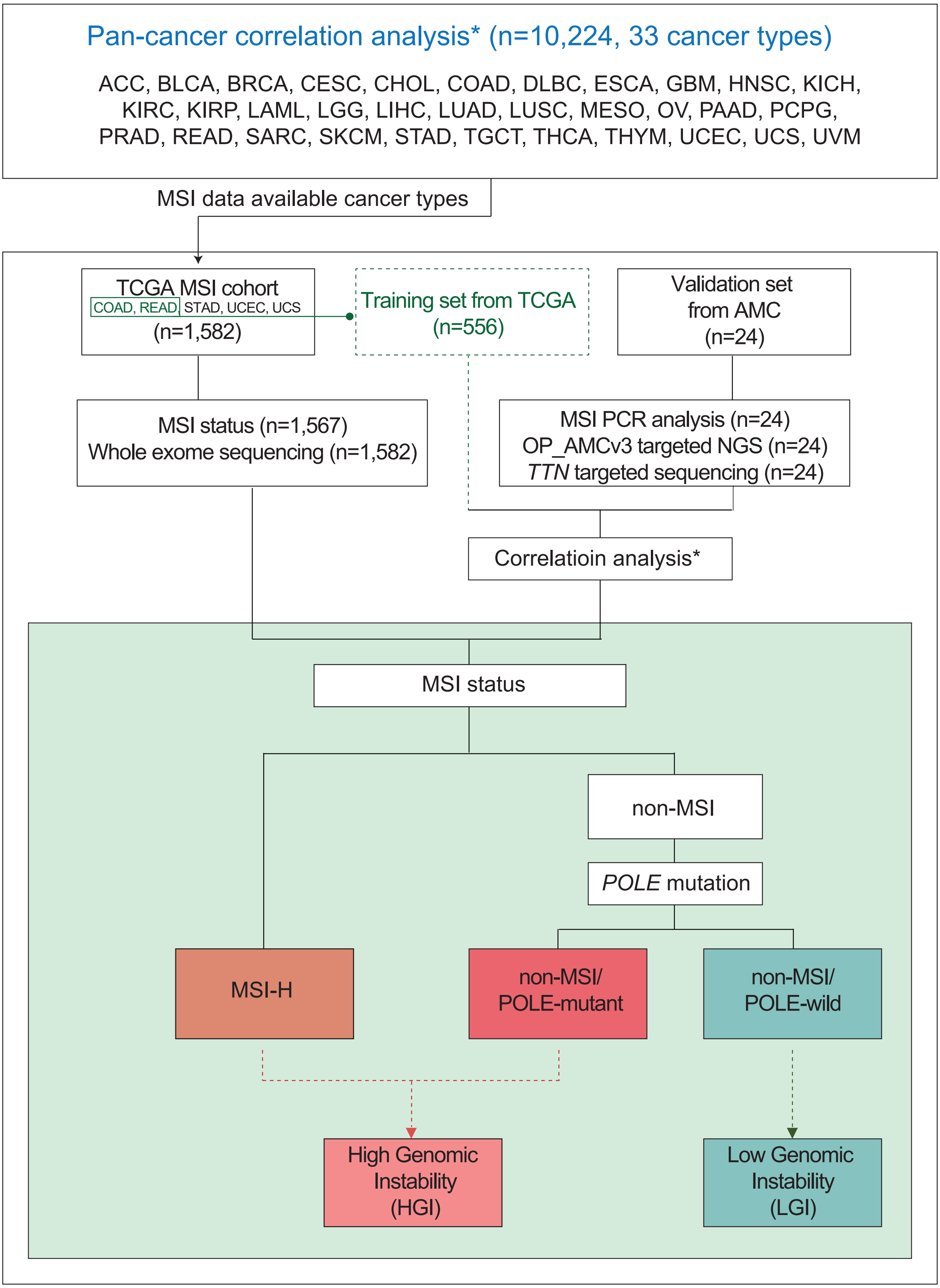

Supplementary Figure 3. Overall study design. Detailed correlation analysis (*) procedures are described in Supplementary Figure 4.

Supplementary Figure 4

Correlation analysis

1st step. Generate Genes x Samples matrix.

	Sample #1	Sample #2	···	Sample #n
Gene #1				
Gene #2				
⋮				
Gene #m				
Total mutation count				

2nd step. Evaluate correlation, and repeat for all genes.

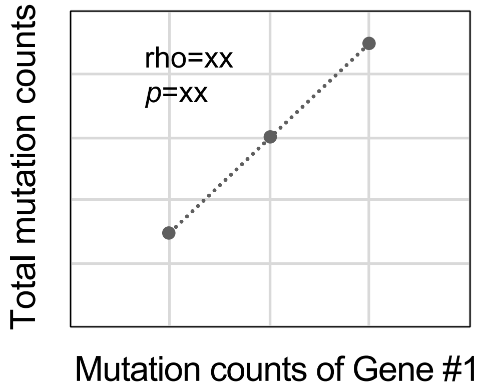

3rd step. Summarize rho and *p* for m genes.

	rho	*p*
Gene #1	xx	xx
Gene #2		
:		
Gene #m		

Supplementary Figure 4. Detailed procedure of the correlation analysis.

Supplementary Table 1. Overview of POLE and mismatch repair related genes mutation profiles.

Patient	Sample id	Gender	Tumor	Age (Diagnosis)	MSI	Three class	Two class	POLE/POLD1 nonSyn variant	POLE/POLD1 nonSyn variant	%	SIFT	PolyPhen	MMR noSyn variants	%	SIFT	PolyPhen2
p-611	SMP000000061	F	Mucinous Adenocarcinoma	61	MSI-H	MSI-H	HGI	–	–							
p-612	SMP0000000612	F	Adenocarcinoma	45	MSS	non-MSI/POLE-wild	LGI	–	–							
p-613	SMP0000000613	M	Adenocarcinoma	60	MSI-H	MSI-H	HGI	POLE c.1625delG	p.Gly542AlafsTer21	24%			MLH1 c.1086delC	15%		
													MLH1 c.1966T>G	42%	deleterious(0)	probably_damaging(0.984)
													PMS2 c.1999G>T	19%		
p-614	SMP0000000614	F	Adenocarcinoma	41	MSI-H	MSI-H	HGI	POLE c.95T>G	p.Leu32Arg	56%	tolerated(0.48)	benign(0.09)	MSH6 c.946delA	41%		
p-615	SMP0000000615	M	Adenocarcinoma	61	MSI-H	MSI-H	HGI	–	–							
p-616	SMP0000000616	M	Adenocarcinoma	69	MSS	non-MSI/POLE-wild	LGI	–	–							
p-617	SMP0000000617	M	Mucinous Adenocarcinoma	32	MSI-H	MSI-H	HGI	POLE c.2683G>A	p.Ala895Thr	3%	tolerated(0.14)	probably_damaging(0.969)	MSH2 c.251delA	23%		
													MSH2 c.1721A>C	47%	deleterious(0)	probably_damaging(1)
													MSH6 c.3261dupC	2%		
p-618	SMP0000000618	M	Adenocarcinoma	48	MSI-H	MSI-H	HGI	–	–				MSH2 c.252T>G	14%	tolerated(0.1)	possibly_damaging(0.517)
													MSH6 c.873_874delCA	71%		
p-619	SMP0000000619	F	Adenocarcinoma	53	MSS	non-MSI/POLE-wild	LGI	–	–				MSH2 c.505A>G	44%	tolerated(0.6)	benign(0.033)
p-620	SMP0000000620	F	Mucinous Adenocarcinoma	63	MSI-H	MSI-H	HGI	–	–				MSH6 c.3261dupC	29%		
													MSH6 c.3656C>T	32%	deleterious(0)	probably_damaging(0.999)
p-621	SMP0000000621	F	Adenocarcinoma	21	MSI-H	MSI-H	HGI	POLE c.1186delG	p.Glu396SerfsTer16	35%			MSH6 c.877C>A	4%		benign(0.11)
p-622	SMP0000000622	F	Adenocarcinoma	55	MSS	non-MSI/POLE-wild	LGI	–	–							
p-623	SMP0000000623	F	Adenocarcinoma	56	MSI-H	MSI-H	HGI	–	–							
p-624	SMP0000000624	F	Adenocarcinoma	80	MSI-H	MSI-H	HGI	–	–							
p-625	SMP0000000625	F	Adenocarcinoma	58	MSS	non-MSI/POLE-wild	LGI		–							
p-626	SMP0000000626	M	Adenocarcinoma	46	MSS	non-MSI/POLE-mutant	HGI	POLE c.857C>G	p.Pro286Arg	25%	deleterious(0)	probably_damaging(1)	MSH2 c.1540G>A	24%	deleterious(0)	possibly_damaging(0.593)
													MSH2 c.1738G>T	29%		
													MSH2 c.2245G>T	27%		
													MSH2 c.2427G>T	4%	tolerated(1)	benign(0.004)
p-627	SMP0000000627	M	Adenocarcinoma	50	MSI-H	MSI-H	HGI	POLE c.4335_4338delTGT(	p.Cys1445TrpfsTer7	3%			MLH1 c.19delG	77%		
p-628	SMP0000000628	M	Adenocarcinoma	58	MSS	non-MSI/POLE-wild	LGI	–	–							
p-629	SMP0000000629	M	Adenocarcinoma	53	MSI-H	MSI-H	HGI	POLE c.292T>G	p.Leu98Val	50%	tolerated(0.33)	benign(0.264)	PMS2 c.1239delA	5%		
													PMS2 c.18C>G	23%	olerated_low_confidence(0.	benign(0.001)
p-630	SMP0000000630	M	Adenocarcinomas (x2)	68	MSS	non-MSI/POLE-wild	LGI	–	–				MSH2 c.505A>G	40%	tolerated(0.6)	benign(0.033)
													ISH6 c.4068_4071dupGAT	58%		
p-631	SMP0000000631	F	Adenocarcinoma	45	MSI-H	MSI-H	HGI	POLE c.6088delG	p.Ala2030ProfsTer18	32%			MLH1 c.2249A>G	5%	deleterious(0.01)	possibly_damaging(0.658)
p-632	SMP0000000632	M	Adenocarcinoma	62	MSS	non-MSI/POLE-wild	LGI	–	–							
p-633	SMP0000000633	F	Adenocarcinoma	46	MSI-H	MSI-H	HGI	POLE c.6145A>G	p.Thr2049Ala	4%	tolerated(0.45)	benign(0.006)	MSH6 c.3261dupC	24%		
								POLE c.1534G>A	p.Ala512Thr	12%	deleterious(0)	possibly_damaging(0.631)	MLH1 c.1900G>T	5%	deleterious(0)	probably_damaging(1)
p-634	SMP0000000634	F	Adenocarcinoma	69	MSS	non-MSI/POLE-wild	LGI	–	–							

Supplementary Table 2. Comparison between TTN sequencing and targeted gene panel sequencing

	TTN sequencing	Targeted sequencing (OncoPanel AMC v3)
**Cost**		
	300 USD (352,850 KRW)	300 USD (352,850 KRW)
**Size**		
Target territory (base pair)	107,976	903,542
Total reads (mean ± SD)	2,422,547 ± 430,671.9	9,015,647 ± 1,205,894
Mean target coverage (mean ± SD)	296.7652 ± 102.4071	165.5416 ± 42.42927)
Percent target bases of 100X (mean ± SD)	0.953154 ± 0.1122055	0.7386273 ± 0.1345896
**Time**		
From raw fastq to final bam (8 cores used in mapping)	5m 35s ± 58s	9m 7s ± 1m 21s
Variant calling	44s ± 12s	3m 20s ± 42s

SD, standard deviation

Supplementary Table 3. The 33 cancer types from TCGA.

	Cancer types	Disease name	No. of patients	MSI status information	MSI status without NA, indeterminiate	Note
1	ACC	Adrenocortical carcinoma	92			
2	BLCA	Bladder urothelial carcinoma	411			
3	BRCA	Breast invasive carcinoma	1,020			
4	CESC	Cervical and endocervical cancers	289			
5	CHOL	Cholangiocarcinoma	36			
6	COAD	Colon adenocarcinoma	406	yes	402	CRC
7	DLBC	Lymphoid neoplasm diffuse large B-cell lymphoma	37			
8	ESCA	Esophageal carcinoma	184			
9	GBM	Glioblastoma multiforme	393			
10	HNSC	Head and neck squamous cell carcinoma	507			
11	KICH	Kidney chromophobe	66			
12	KIRC	Kidney renal clear cell carcinoma	369			
13	KIRP	Kidney renal papillary cell carcinoma	281			
14	LAML	Acute myeloid leukemia	141			
15	LGG	Brain lower grade glioma	512			
16	LIHC	Liver hepatocellular carcinoma	363			
17	LUAD	Lung adenocarcinoma	567			
18	LUSC	Lung squamous cell carcinoma	485			
19	MESO	Mesothelioma	82			
20	OV	Ovarian serous cystadenocarcinoma	412			
21	PAAD	Pancreatic adenocarcinoma	177			
22	PCPG	Pheochromocytoma and paraganglioma	179			
23	PRAD	Prostate adenocarcinoma	497			
24	READ	Rectum adenocarcinoma	150	yes	150	CRC
25	SARC	Sarcoma	236			
26	SKCM	Skin cutaneous melanoma	466			
27	STAD	Stomach adenocarcinoma	439	yes	439	
28	TGCT	Testicular germ cell tumors	145			
29	THCA	Thyroid carcinoma	492			
30	THYM	Thymoma	123			
31	UCEC	Uterine corpus endometrial carcinoma	530	yes	520	
32	UCS	Uterine carcinosarcoma	57	yes	56	
33	UVM	Uveal melanoma	80			
			10,224		1,567	

Supplementary Data 1. Correlation coefficient of all tested genes for each tumor type.

Supplementary Data 2. The most highly mutated genes for each tumor type.

Supplementary Data 3. Result of correlation analysis in TCGA colorectal cancer (CRC) cohort.
